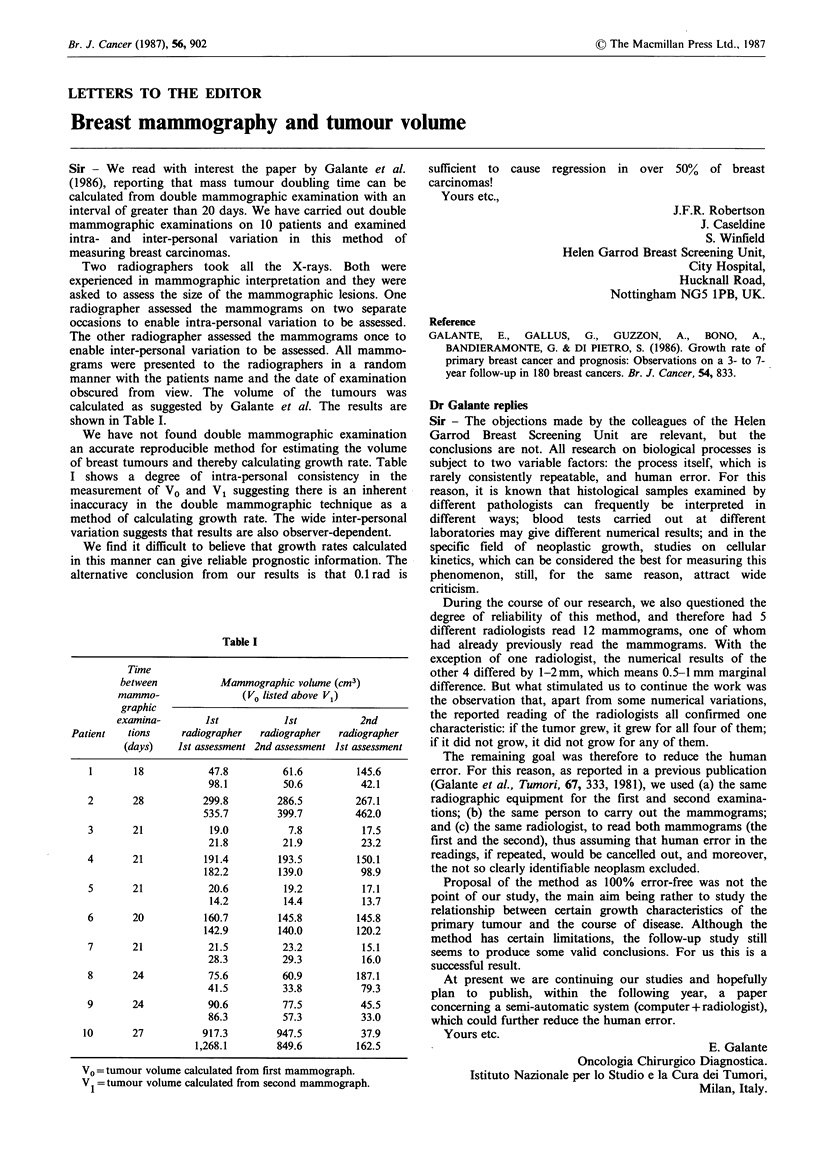# Breast mammography and tumour volume.

**DOI:** 10.1038/bjc.1987.309

**Published:** 1987-12

**Authors:** J. F. Robertson, J. Caseldine, S. Winfield


 © The Macmillan Press Ltd., 1987

### LETTERS TO THE EDITOR

### Breast mammography and tumour volume

**Sir** – We read with interest the paper by Galante *et al.* (1986), reporting that mass tumour doubling time can be calculated from double mammographic examination with an interval of greater than 20 days. We have carried out double mammographic examinations on 10 patients and examined intra- and inter-personal variation in this method of measuring breast carcinomas.

Two radiographers took all the X-rays. Both were experienced in mammographic interpretation and they were asked to assess the size of the mammographic lesions. One radiographer assessed the mammograms on two separate occasions to enable intra-personal variation to be assessed. The other radiographer assessed the mammograms once to enable inter-personal variation to be assessed. All mammograms were presented to the radiographers in a random manner with the patients name and the date of examination obscured from view. The volume of the tumours was calculated as suggested by Galante *et al.* The results are shown in Table I.

We have not found double mammographic examination an accurate reproducible method for estimating the volume of breast tumours and thereby calculating growth rate. Table I shows a degree of intra-personal consistency in the measurement of $V_0$ and $V_1$ suggesting there is an inherent inaccuracy in the double mammographic technique as a method of calculating growth rate. The wide inter-personal variation suggests that results are also observer-dependent.

We find it difficult to believe that growth rates calculated in this manner can give reliable prognostic information. The alternative conclusion from our results is that 0.1 rad is sufficient to cause regression in over 50% of breast carcinomas!

Yours etc.,

J.F.R. Robertson
J. Caseldine
S. Winfield
Helen Garrod Breast Screening Unit,
City Hospital,
Hucknall Road,
Nottingham NG5 1PB, UK.

**Table I**

| Patient | *Time between mammographic examinations (days)* | *Mammographic volume (cm³) ($V_0$ listed above $V_1$)* 1st radiographer 1st assessment | 1st radiographer 2nd assessment | 2nd radiographer 1st assessment |
|---|---|---|---|---|
| 1 | 18 | 47.8 | 61.6 | 145.6 |
| | | 98.1 | 50.6 | 42.1 |
| 2 | 28 | 299.8 | 286.5 | 267.1 |
| | | 535.7 | 399.7 | 462.0 |
| 3 | 21 | 19.0 | 7.8 | 17.5 |
| | | 21.8 | 21.9 | 23.2 |
| 4 | 21 | 191.4 | 193.5 | 150.1 |
| | | 182.2 | 139.0 | 98.9 |
| 5 | 21 | 20.6 | 19.2 | 17.1 |
| | | 14.2 | 14.4 | 13.7 |
| 6 | 20 | 160.7 | 145.8 | 145.8 |
| | | 142.9 | 140.0 | 120.2 |
| 7 | 21 | 21.5 | 23.2 | 15.1 |
| | | 28.3 | 29.3 | 16.0 |
| 8 | 24 | 75.6 | 60.9 | 187.1 |
| | | 41.5 | 33.8 | 79.3 |
| 9 | 24 | 90.6 | 77.5 | 45.5 |
| | | 86.3 | 57.3 | 33.0 |
| 10 | 27 | 917.3 | 947.5 | 37.9 |
| | | 1,268.1 | 849.6 | 162.5 |

$V_0$ = tumour volume calculated from first mammograph.
$V_1$ = tumour volume calculated from second mammograph.

**Reference**

GALANTE, E., GALLUS, G., GUZZON, A., BONO, A., BANDIERAMONTE, G. & DI PIETRO, S. (1986). Growth rate of primary breast cancer and prognosis: Observations on a 3- to 7-year follow-up in 180 breast cancers. *Br. J. Cancer*, **54**, 833.